# Three Novel Herpesviruses of Endangered *Clemmys* and *Glyptemys* Turtles

**DOI:** 10.1371/journal.pone.0122901

**Published:** 2015-04-15

**Authors:** Robert J. Ossiboff, Bonnie L. Raphael, Alyssa D. Ammazzalorso, Tracie A. Seimon, Alisa L. Newton, Tylis Y. Chang, Brian Zarate, Alison L. Whitlock, Denise McAloose

**Affiliations:** 1 Wildlife Conservation Society, Zoological Health Program, Bronx, New York, United States of America; 2 Fordham University, Bronx, New York, United States of America; 3 Albert Einstein College of Medicine of Yeshiva University, Bronx, New York, United States of America; 4 New Jersey Division of Fish and Wildlife, Endangered and Nongame Species Program, Clinton, New Jersey, United States of America; 5 U. S. Fish and Wildlife Service, Hadley, Massachusetts, United States of America; University of Liverpool, UNITED KINGDOM

## Abstract

The rich diversity of the world’s reptiles is at risk due to significant population declines of broad taxonomic and geographic scope. Significant factors attributed to these declines include habitat loss, pollution, unsustainable collection and infectious disease. To investigate the presence and significance of a potential pathogen on populations of critically endangered bog turtles (*Glyptemys muhlenbergii*) as well sympatric endangered wood (*G*. *insculpta*) and endangered spotted (*Clemmys guttata*) turtles in the northeastern United States, choanal and cloacal swabs collected from 230 turtles from 19 sites in 5 states were screened for herpesvirus by polymerase chain reaction. We found a high incidence of herpesvirus infection in bog turtles (51.5%; 105/204) and smaller numbers of positive wood (5) and spotted (1) turtles. Sequence and phylogenetic analysis revealed three previously uncharacterized alphaherpesviruses. Glyptemys herpesvirus 1 was the predominant herpesvirus detected and was found exclusively in bog turtles in all states sampled. Glyptemys herpesvirus 2 was found only in wood turtles. Emydid herpesvirus 2 was found in a small number of bog turtles and a single spotted turtle from one state. Based on these findings, Glyptemys herpesvirus 1 appears to be a common infection in the study population, whereas Glyptemys herpesvirus 2 and Emydid herpesvirus 2 were not as frequently detected. Emydid herpesvirus 2 was the only virus detected in more than one species. Herpesviruses are most often associated with subclinical or mild infections in their natural hosts, and no sampled turtles showed overt signs of disease at sampling. However, infection of host-adapted viruses in closely related species can result in significant disease. The pathogenic potential of these viruses, particularly Emydid herpesvirus 2, in sympatric chelonians warrants additional study in order to better understand the relationship of these viruses with their endangered hosts.

## Introduction

The non-avian reptiles, defined as all members of the orders Crocodylia (alligators and crocodiles), Lepidosaura (snakes and lizards), Sphenodontia (tuatara), and Testudines (tortoises and turtles) and hereafter referred to as reptiles, are a large and diverse group of vertebrates adapted to a wide range of terrestrial, marine and freshwater environments. With over 10,000 described species, they are the second-most diverse group of tetrapods after birds [1]. However significant population declines of broad taxonomic and geographic scope threaten this rich diversity. A recent global analysis of extinction risk in reptiles found that nearly one in five species is threatened with extinction, with the highest proportion of threatened species residing in freshwater environments, tropical regions and on oceanic islands [2]. Factors contributing to reptile declines include loss and degradation of habitat, environmental pollution, climate change, unsustainable collection, and infectious disease [3,4].

While sporadic outbreaks of infectious disease have been associated with mortalities in a variety of reptile species, infectious disease appears to be a particularly important contributor to population decline in chelonians (turtles, tortoises and terrapins) [4–7]. The most extensively documented and arguably the most globally significant pathogens of chelonians are enveloped, double-stranded viruses in the family Herpesviridae. In the green sea turtle (*Chelonia mydas*), a species listed as endangered by the International Union for the Conservation of Nature (IUCN), a herpesvirus is the causative agent of epizootics of epidermal necrosis (“grey patch disease”) [8,9]. A similar herpesvirus is also the cause of epidermal necrosis in endangered loggerhead turtles (*Caretta caretta*) [10]. Lung-eye-and-trachea virus (LETV), another herpesvirus of green sea turtles, is the causative agent of epizootics of pneumonia and necrotizing oral and upper respiratory inflammation [11]. In all species of sea turtle, fibropapilloma-associated turtle herpesvirus infection can result in the formation of cutaneous, papillary tumors that result in significant morbidity and often mortality [12–15].

Herpesviruses are also important disease agents in terrestrial and freshwater chelonians. Fatal systemic disease with characteristic herpesviral-like inclusions has been observed in Pacific pond turtles (*Actinemys marmorata*), painted turtles (*Chrysemys picta*), and map turtles (*Graptemys spp*.) [16–18]. A herpesvirus (Terrapene herpesvirus 1; TeHV-1) of eastern box turtles (*Terrapene carolina carolina*) has been identified in animals with either fatal systemic disease or concurrent ranavirus infection [19]. Emydid herpesvirus 1 (EmyHV-1) was described in an eastern river cooter (*Pseudemys concinna concinna*) that died suddenly [20]. Molecular or histologic evidence of herpesviral infection has also been associated with significant oral inflammation in a number of tortoise species [9,21–23].

Turtles of the genera *Clemmys* and *Glyptemys* are semiaquatic, freshwater turtles of the family Emydidae. The genus *Glyptemys* is composed of two species, the critically endangered bog turtle (*G*. *muhlenbergii*) [24] and the endangered wood (*G*. *insculpta*) turtle [25]. Native to the eastern United States, bog turtles occur in disjunct populations that inhabit primarily spring-fed wetlands, fens, and wet meadow habitats. Through the twentieth century, bog turtle habitat and population numbers are estimated to have decreased over 80% with an estimated 50% decline in range and numbers between 1977–1997 alone [24]. Wood turtles are native to streams, rivers and adjoining forest of the northeastern and Great Lakes region of the United States and southeastern Canada. Estimates are that half (50%) of the wood turtle population has been lost over the past 100 years [25]. The sole member of the genus *Clemmys* is the spotted turtle (*C*. *guttata*). It inhabits a variety of types of wetland in the eastern United States and the Great Lakes region of Canada and the United States. Like the wood turtle, *C*. *guttata* is also classified as endangered by the IUCN [26].

Over the past 10 years, increased mortality rates have been observed in some populations of bog turtles in the northeastern United States. Due to the significant role that herpesvirus play around the world in turtle health and mortality, this study investigated the potential for herpesviruses to serve as pathogens in critically endangered bog turtles and sympatric endangered wood and spotted turtles in the northeastern United States. Results of this study identified three novel herpesviruses, with a virus unique to each *Glyptemys* species, and a third herpesvirus identified in both *G*. *muhlenbergii* and *C*. *guttata*. No sampled turtles showed outward evidence of clinical disease. While the significance of the identified viruses to the individual and overall population health of these three endangered species is unclear, documentation of these viruses provides important baseline information for future management and conservation efforts.

## Materials and Methods

### Ethics Statement

All procedures involving animals were approved by and coordinated with the United States Fish and Wildlife Service Northeast Region Endangered Species Division, Massachusetts Division of Fisheries and Wildlife, Delaware Division of Fish and Wildlife, New Jersey Division of Fish, Game and Wildlife, New York State Division of Fish, Wildlife and Marine Resources, and Pennsylvania Fish and Boat Commission. Trained biologists were primarily responsible for locating and collecting the turtles. Veterinary personnel performed sample collection and health assessments of the turtles.

### Sampling

During the springs of 2011, 2013, and 2014 health assessments were performed in populations of bog turtles selected by biologists as representative of healthy populations as well as populations of concern due to recent mortality events. Most turtles were located in fens by walking the areas and gently probing the soft substrate or by direct visualization of animals. Turtles then were manually restrained and the choana and then cloaca were swabbed with a single rayon-tipped, plastic shaft, fine tip swab (Medical Wire and Equipment, Wiltshire, England). Samples were collected from the states of Massachusetts (2 sites; April and May, 2011), New York (2 sites; May, 2011), New Jersey (10 sites; May, 2013 and 2014), Pennsylvania (3 sites; May, 2013), and Delaware (2 sites; May, 2013 and 2014). Swabs were placed in 2ml polypropylene micro tubes with screw caps (Sarstedt, Nümbrecht, Germany), then stored at 4° C for up to 48 hours and at -80° C for long term storage. The total sample population (*n* = 230) included bog (*n =* 204), spotted (*n =* 17) and wood (*n =* 9) turtles (Table 1).

**Table 1:** Species list, geographic distribution and proportion of PCR herpesvirus positive animals in study population, and virus-host association.

**Species**	**PCR positive / Number tested**					**Total**	**Virus**
	**DE**	**PA**	**NJ**	**NY**	**MA**		
Bog turtle (*Glyptemys muhlenbergii*)	20/44	6/18	61/99	11/19	7/24	105/204	GlyHV-1, EmyHV-2
Wood turtle (*Glyptemys insculpta*)	-	3/4	2/5	-	-	5/9	GlyHV-2
Spotted turtle (*Clemmys guttata*)	0/4	0/8	1/5	-	-	1/17	EmyHV-2

### Nucleic acid extraction, Herpesvirus PCR and DNA sequencing

Swab nucleic acid extraction was performed using either the QIAamp DNA Mini Kit (Qiagen Inc., Valencia, California, USA) or Prepman Ultra (Applied Biosystems, Foster City, California, USA) per the manufacturers’ recommendations. Quantification of extracted nucleic acids was performed on a subset of samples using Qubit fluorometric quantification (Invitrogen, Carlsbad, California, USA). Qualitative, nested PCR amplification of a short, 181 bp fragment of the herpesviral DNA-dependent DNA polymerase (hereafter referred to as DNA polymerase) was performed using previously described methods [27]. Reactions were prepared to contain the following: 12.5 μl of Amplitaq Gold 360 Master Mix (Applied Biosystems, Foster City, California, USA), 25 pmol each of degenerate primers DFA, ILK and KG1 (round 1) or TGV and IYG (round 2), 2 μl of either the nucleic acid extract (round 1) or the round 1 reaction product (round 2), and molecular-grade water to bring the total reaction volume to 25 μl. Reaction conditions were: 95° C for 12 m; 45 cycles at 95° C for 20 s, 46° C for 60 s, and 72° C for 60 s; and a final extension step of 72° C for 10 m. A commercially prepared plasmid containing herpesvirus primer binding sites was used as a positive control. Amplified PCR products were visualized with gel electrophoresis and SYBR Safe DNA Gel Stain (Invitrogen, Carlsbad, CA, USA). Amplicons of approximately 200 bp were treated with ExoSAP-IT enzymatic cleanup reagent (Affymetrix, Cleveland, Ohio, USA) and submitted for commercial sequencing (Genewiz, Inc., South Plainfield, New Jersey, USA) using primers TGV and IYG. DNA sequences were edited, trimmed and aligned using Geneious bioinformatics software (version 6.1.7; Biomatters, Ltd., Auckland, New Zealand). Distance matrix analysis was performed using Geneious, and sequence analysis was performed using the basic local alignment search tool (BLAST) in Genbank (National Center for Biotechnology information) [28]. To obtain additional sequence of the DNA polymerase gene, two altered second round PCR reactions were performed using primers DFA and IYG or TGV and KG1, resulting in amplicons of approximately 500 and 430 bp, respectively. Samples were enzymatically treated submitted for sequencing as described above. DNA sequences were edited, trimmed, assembled into a single 689 bp fragment, aligned and analyzed as detailed above. Sequences were uploaded to Genbank (accessions KM357867-KM357869). The amplified short, 181 bp polymerase fragments correspond to nucleotides 299–480 of the Genbank accessions.

### Phylogenetic analysis

Deduced amino acid sequences of the 689 bp fragment of the partial DNA polymerase gene were aligned with other members of the Herpesviridae reported in Genbank ranging from 58–231 residues in length (S1 Table) using Geneious bioinformatics software (version 6.1.7). Bayesian analysis of the amino acid alignment was performed using a MrBayes plugin for Geneious, with a fixed poisson rate matrix, gamma distributed rate variation, 4 heated chains, unconstrained branch lengths and a subsampling frequency of 200 [29]; the first 25% of 1,100,000 chains were discarded as burn-in. Maximum likelihood (ML) analysis of the alignment was performed using the PHYML plugin for Geneious utilizing the WAG substitution model, estimated gamma distribution parameter and proportion of invariant sites and bootstrap branch support with 1000 subsets [30]. Phylogenetic trees were visualized using FigTree software (http://tree.bio.ed.ac.uk/software/figtree/).

## Results

Overall, 111 of 230 (48.3%) turtles were positive for herpesvirus by PCR. Positive results were obtained from 105 of 204 (51.5%) bog turtles, 5 of 9 wood turtles (55.6%), and 1 of 17 spotted turtles (5.9%; Table 1). Sequencing of the PCR DNA polymerase short amplicon yielded a 181 bp product (after trimming of primer sequence) for all PCR positive turtles, and sequence alignments revealed three distinct polymerase sequences with 87.3–90.6% similarity to each other. Using two modified second round reactions of the nested PCR, a 689 bp fragment of the DNA polymerase was assembled for one representative sample for each virus; the larger polymerase fragments were 89.3–91.7% identical to each other.

The predominant herpesvirus identified was found exclusively in bog turtles (99 of 204 [48.5%] of all bog turtles tested; 99 of 105 [94.3%] of herpesvirus positive bog turtles) and was present in bog turtle swabs from each of the 5 states. The shorter amplicons for all 99 positive turtles were 99.4–100% identical to each other. Only 8 turtles contained single nucleotide polymorphisms from the consensus sequence, including 6 bog turtles from both sites in MA with a silent C→G mutation (position 374 of Genbank accession KM357867) and two bog turtles from a single site in NJ with a silent C→T mutation (position 356 of accession KM357867). BLAST analysis of the large polymerase fragment revealed 90% sequence identity to TeHV 1 (KJ004665), 75% to LETV (EU006876) and 73% to Tortoise herpesvirus (TortHV; AB047545). We hereafter refer to this virus in the manuscript as Glyptemys herpesvirus 1 (GlyHV-1).

A second herpesvirus sequence was found exclusively in wood turtles and was present in 5 of the 9 (55.6%) wood turtles tested. The short polymerase amplicons of all three positive turtles were 100% identical to each other. Blast analysis of the large polymerase fragment revealed 89% sequence identity to GlyHV-1, 87% to TeHV-1 (KJ004665), 74% to LETV (EU006876) and 73% to TortHV (AB047545); we hereafter refer to this virus in the manuscript as Glyptemys herpesvirus 2 (GlyHV-2).

A third herpesvirus sequence was present in a minority of herpesvirus positive bog turtles (6 of 204 [2.9%] of all bog turtles tested; 6 of 105 [5.7%] PCR positive bog turtles) and was also found in 1 of 17 (5.9%) spotted turtles. All turtles positive for this virus were found in New Jersey, with all positive bog turtles residing at a site distinct from the positive spotted turtle. The short polymerase fragments in all four positive turtles were 100% identical to each other. Blast analysis of the large polymerase fragment revealed 92% sequence identity to GlyHV-1, 90% to GlyHV-2, 91% to TeHV 1 (KJ004665), 73% to LETV (EU006876) and 73% to TortHV (AB047545); we hereafter refer to this virus in the manuscript as Emydid herpesvirus 2 (EmyHV-2).

**Fig 1:**
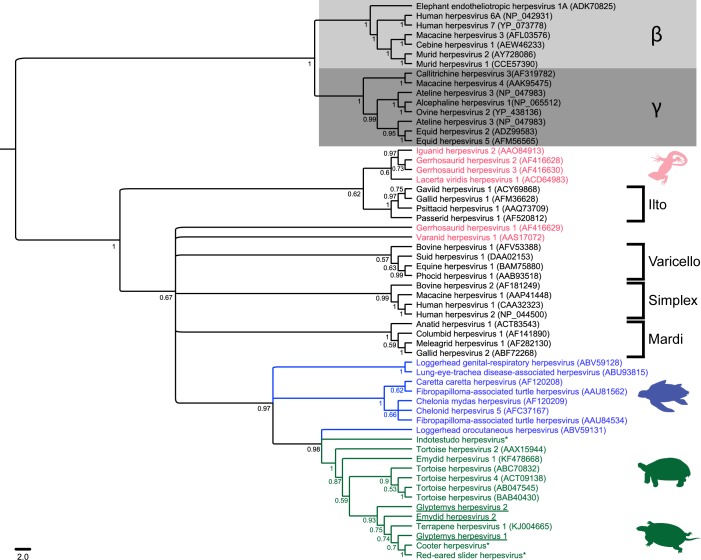
Midpoint-rooted Bayesian phylogenetic tree of predicted amino acid sequences of herpesviral DNA-dependent DNA polymerases. Bayesian posterior probabilities are shown at branch points. Glyptemys herpesvirus 1 and 2 and Emydid herpesvirus 2 are underlined. Herpesviruses of freshwater and terrestrial turtles and tortoises are shown in green; herpesviruses of marine turtles are shown in blue and other reptile herpesviruses are shown in red. Brackets demarcate *Alphaherpesvirinae* genera *Ilto-*, *Varicello-*, *Simplex* and *Mardiviridae*. The *Betaherpesvirinae* are highlighted by a light grey box, and the *Gammaherpesvirinae* by a dark grey box. Genbank accession numbers are shown in parentheses following the virus name. Sequences not published in Genbank are marked with an asterisk (*).

Bayesian (Fig. 1) and ML (data not shown) phylogenetic analysis placed all three viruses within the subfamily *Alphaherpesvirinae*, clustering most closely with representatives of the genus *Scutavirus* with a 97% Bayesian posterior probability value and a 69% ML bootstrap value. All three viruses (underlined, Fig. 1) clustered together in a monophyletic group that fell within a clade containing other herpesviruses of freshwater and terrestrial turtles and tortoises (green, Fig. 1) and a single marine turtle herpesvirus (blue, Fig. 1), Loggerhead orocutaneous herpesvirus (ABV59131).

## Discussion

The identification of known and novel pathogens in wildlife is critical in understanding factors contributing to population declines. Herpesviruses are significant pathogens in many species, though the ecology of infection and disease in many remains to be defined. Through sequence and phylogenetic analysis of a portion of the viral DNA polymerase gene, we identified three novel viruses in critically endangered bog turtles and endangered spotted and wood turtles that were overtly healthy. These findings represent the first identification and partial characterization of a herpesvirus in each of these species, and nearly doubles the total number of herpesviruses (*n* = 7) with DNA sequence characterization from emydid turtles [19,20].

All reptile herpesviruses that have been genetically characterized to date are most closely related to members of the subfamily *Alphaherpesvirinae* [10,31–33]. The International Committee on the Taxonomy of Viruses formally recognizes two herpesviruses of chelonians, Chelonid herpesviruses 5 and 6. Chelonid herpesvirus 5, which includes the causative agents of “grey patch disease” in green sea turtles, epidermal necrosis in loggerhead sea turtles, and virus associated-fibropapillomas in sea turtles, is the type species of the alphaherpesvirus genus, *Scutavirus* [34]. While only two chelonid herpesviruses are formally recognized by the ICTV, a substantial number of herpesviruses have been described from turtles and tortoises (Fig. 1, S1 Table). Previous phylogenetic analyses have grouped all chelonian herpesviruses within a single, monophyletic clade in the *Alphaherpesvirinae* that contains the type species of the genus *Scutavirus* [10,19,32]. Our results were consistent with the previous studies, but also suggest that herpesviruses of freshwater and terrestrial turtles and tortoises (highlighted with green text in Fig. 1) are more closely related to each other than to herpesviruses of marine turtles (highlighted in blue text). As the number of chelonian viruses characterized increases with time and continued study, it may become apparent that a single genus for all chelonian herpesviruses is not sufficient and the creation of separate genera for viruses of marine and terrestrial/freshwater turtles would be more appropriate.

Herpesvirus-associated disease is commonly reported in domestic and wild animal species as well as in humans. It is widely recognized that herpesvirus infection in a natural, immunocompetent host is frequently subclinical or mild and is often followed by viral latency, presumably due to co-evolution of the virus and host [35]. GlyHV-1 was found in 54% (68/126) of sampled bog turtles across 5 states (New Jersey, Pennsylvania, Delaware, New York and Massachusetts) that together represent a significant portion of the natural range of this species in the northeastern United States. Given the high incidence of GlyHV-1 in the study population without obvious evidence of morbidity upon physical examination at the time of sampling, it is likely that GlyHV-1 is a host-adapted pathogen in this species and is of limited clinical significance in healthy individuals and populations. Additionally, opportunistic necropsies were performed on 15 bog turtles found dead within or adjacent to the study sites. While postmortem autolysis and scavenging of target tissues precluded interpretation in 8 animals, no evidence of viral cytopathic effect or herpesviral-like inflammatory disease was noted in the remaining animals. However, clinically significant disease associated with either recrudescence of a latent infection or de novo infection could produce significant disease in the face of stress and associated immunosuppression [36,37]. The relationship between GlyHV-2 and wood turtles is likely similar, with the virus being found in 5 of the 9 wood turtles sampled but not in bog or spotted turtles.

In contrast to the high incidence of GlyHV-1 in bog turtles, Emydid herpesvirus 2 was found in only a small fraction of the tested bog turtles (2.9%), and all positive animals were from one site in New Jersey. Unexpectedly, EmyHV-2 was also found in one of the 17 (5.9%) spotted turtles from a neighboring site in the same state. Unlike the mild infections often seen in herpesvirus infections of a natural host, severe or fatal infection have been documented on multiple occasions when a herpesvirus of one species infects a closely related species [38–41]. In emydid turtles in particular, cross species transmission of a herpesvirus has been speculated to result in fatal disease [18]. While additional surveillance of turtles in this region is required to interpret the significance of this finding, it is possible that EmyHV-2 is a clinically significant pathogen of either spotted or bog turtles or, possibly, both.

In summary, we identified three novel alphaherpesviruses of endangered *Glyptemys* and *Clemmys* turtles. GlyHV-1 appears to be a common infection in wild bog turtles of unknown clinical significance. Despite a small sample size, GlyHV-2 was common in sampled wood turtles. The incidence of EmyHV-2 was low in both bog and spotted turtles, but given the pathogenic potential of host-adapted herpesvirus infections in closely related species, EmyHV-2 may be a significant pathogen of either bog or spotted turtles. Accordingly, care should be taken when working with the two species in the wild or in captivity to limit potential virus exposure. Additional studies to further characterize the distribution and incidence of EmyHV-2 in spotted turtles may be helpful in better understanding the significance of this virus to the health of the hosts.

## Supporting Information

S1 Table Additional sequences used for phylogenetic analysis. (DOCX)
