# Supplementary material for: Three Novel Herpesviruses of Endangered Clemmys and Glyptemys Turtles
Source: PLoS One. 2015 Apr 15;10(4):e0122901. doi: 10.1371/journal.pone.0122901 (PMC4398433; doi:10.1371/journal.pone.0122901)
Supplement: S1 Table — (DOCX) [file pone.0122901.s001.docx]

Supplemental Table 1: Additional sequences used for phylogenetic analysis

| **Subfamily** | **Genus** | **Name** | **Genbank Accession** | **Length (AA)** | **Residues (AA)** |
| --- | --- | --- | --- | --- | --- |
| *Alphaherpesvirinae* | *Iltovirus* | Gallid herpesvirus 1 | AFM36628 | 229 | 640-868 |
|  |  | Gaviid herpesvirus 1 | ACY69868 | 168 | - |
|  |  | Passerid herpesvirus 1 | AF520812 | 161 | - |
|  |  | Psittacid herpesvirus 1 | AAQ73709 | 229 | 638-866 |
|  | *Mardivirus* | Anatid herpesvirus 1 | ACT83543 | 228 | 706-933 |
|  |  | Columbid herpesvirus 1 | AF141890 | 165 | - |
|  |  | Gallid herpesvirus 2 | ABF72268 | 228 | 706-933 |
|  |  | Meleagrid herpesvirus 1 | AF282130_33 | 228 | 691-918 |
|  | *Scutavirus* | Caretta caretta herpesvirus | AF120208 | 61 | - |
|  |  | Chelonia mydas herpesvirus | AF120209 | 61 | - |
|  |  | Chelonid herpesvirus 5 | AFC37167 | 165 | - |
|  |  | Fibropapilloma-associated turtle herpesvirus | AAU81562 | 231 | 323-553 |
|  |  | Fibropapilloma-associated turtle herpesvirus | AAU84534 | 231 | 650-880 |
|  | *Simplexvirus* | Bovine herpesvirus 2 | AF181249 | 228 | 727-954 |
|  |  | Human herpesvirus 1 | CAA32323 | 228 | 725-952 |
|  |  | Human herpesvirus 2 | NP_044500 | 228 | 730-957 |
|  |  | Macacine herpesvirus 1 | AAP41448 | 228 | 730-957 |
|  | *Varicellovirus* | Bovine herpesvirus 1 | AFV53388 | 230 | 758-987 |
|  |  | Equine herpesvirus 1 | BAM75880 | 228 | 726-953 |
|  |  | Phocid herpesvirus 1 | AAB93518 | 171 | - |
|  |  | Suid herpesvirus 1 | DAA02153 | 222 | 581-802 |
|  | *Unclassified (Lizard)* | Gerrhosaurid herpesvirus 1 | AF416629 | 62 | - |
|  |  | Gerrhosaurid herpesvirus 2 | AF416628 | 62 | - |
|  |  | Gerrhosaurid herpesvirus 3 | AF416630 | 62 | - |
|  |  | Lacerta viridis herpesvirus 1 | ACD64983 | 81 | - |
|  | *Unclassified (Turtle)* | Cooter herpesvirus | # | 62 | - |
|  |  | Emydid herpesvirus 1 | KF478668 | 130 | - |
|  |  | Indotestudo herpesvirus | # | 62 | - |
|  |  | Loggerhead genital-respiratory herpesvirus | ABV59128 | 165 | - |
|  |  | Loggerhead orocutaneous herpesvirus | ABV59131 | 229 | 2-230 |
|  |  | Lung-eye-trachea disease-associated herpesvirus | ABU93815 | 165 | - |
|  |  | Red-eared slider herpesvirus | # | 62 | - |
|  |  | Terrapene herpesvirus 1 | KJ004665 | 165 | - |
|  |  | Tortoise herpesvirus | BAB40430 | 229 | 2-230 |
|  |  | Tortoise herpesvirus | ABC70832 | 62 | - |
|  |  | Tortoise herpesvirus | AB047545 | 165 | - |
|  |  | Tortoise herpesvirus 2 | AAX15944 | 70 | - |
|  |  | Tortoise herpesvirus 4 | ACT09138 | 145 | - |
| *Betaherpesvirinae* | *Cytomegalovirus* | Cebine herpesvirus 1 | AEW46233 | 226 | 588-813 |
|  |  | Macacine herpesvirus 3 | AFL03576 | 226 | 599-824 |
|  | *Muromegalovirus* | Murid herpesvirus 1 | CCE57390 | 227 | 633-859 |
|  |  | Murid herpesvirus 2 | AY728086 | 228 | 320-547 |
|  | *Proboscivirus* | Elephant endotheliotropic herpesvirus 1A | ADK70825 | 231 | 619-849 |
|  | *Roseolovirus* | Human herpesvirus 6A | NP_042931 | 227 | 580-806 |
|  |  | Human herpesvirus 7 | YP­_073778 | 227 | 580-806 |
| *Gammaherpesvirinae* | *Lymphocryptovirus* | Callitrichine herpesvirus 3 | AF319782_6 | 230 | 589-818 |
|  |  | Macacine herpesvirus 4 | AAK95475 | 230 | 592-821 |
|  | *Macavirus* | Alcephaline herpesvirus 1 | NP_065512 | 229 | 610-838 |
|  |  | Ovine herpesvirus 2 | YP_438136 | 230 | 581-810 |
|  | *Percavirus* | Equid herpesvirus 2 | ADZ99583 | 226 | 590-815 |
|  |  | Equid herpesvirus 5 | AFM56565 | 227 | 594-820 |
|  | *Rhadinovirus* | Ateline herpesvirus 3 | NP_047983 | 226 | 582-807 |
|  |  | Macacine herpesvirus 5 | NP_570750 | 226 | 596-821 |
| *Unassigned* |  | Iguanid herpesvirus 2 | AAO84913 | 230 | 2-231 |
|  |  | Varanid herpesvirus 1 | AAS17072 | 62 | - |
| # Sequence reported in Sim *et al* (2015) | | | | | |
